# Bidirectional matching and aggregation network for few-shot relation extraction

**DOI:** 10.7717/peerj-cs.1272

**Published:** 2023-03-06

**Authors:** Zhongcheng Wei, Wenjie Guo, Yunping Zhang, Jieying Zhang, Jijun Zhao

**Affiliations:** 1 School of Information and Electrical Engineering, Hebei University of Engineering, Handan, Hebei, China; 2 Hebei Key Laboratory of Security & Protection Information Sensing and Processing, Handan, Hebei, China

**Keywords:** Relation extraction, Few-shot learning, Prototypical network, Knowledge graph, Long-tail distribution

## Abstract

Few-shot relation extraction is used to solve the problem of long tail distribution of data by matching between query instances and support instances. Existing methods focus only on the single direction process of matching, ignoring the symmetry of the data in the process. To address this issue, we propose the bidirectional matching and aggregation network (BMAN), which is particularly powerful when the training data is symmetrical. This model not only tries to extract relations for query instances, but also seeks relational prototypes about the query instances to validate the feature representation of the support set. Moreover, to avoid overfitting in bidirectional matching, the data enhancement method was designed to scale up the number of instances while maintaining the scope of the instance relation class. Extensive experiments on FewRel and FewRel2.0 public datasets are conducted and evaluate the effectiveness of BMAN.

## Introduction

Knowledge graphs have multiple applications in question answering for medical domain (Huang et al., 2021) and data analytics and prediction for Biological domain (Alshahrani, Thafar & Essack, 2021). Nevertheless, the existing Knowledge graphs is incomplete, which limits the scope of knowledge applications. Relation extraction for building knowledge graphs provides indispensable knowledge, which are represented by triplet format <*head entity*, *relation*, *tail entity*>. Different from link prediction (Sun et al., 2019), which uses already existing triples to derive related triples, relation extraction determines the relation between entities in a given text to construct the triples. Relations extraction is generally handled as a text classification task, classifying entity-pair relations into a certain relation class, but differently from text classification which focuses on the text as a whole (Jia & Wang, 2022), the relation extraction task focuses more on the context of the entity. The study of the extraction of relational facts from a large *corpus* has important implications for the enrichment of the knowledge graph.

Conventional methods of manually labeling data face inefficiencies and expensive costs. Distant supervision learning (Mintz et al., 2009) was proposed to address the above issues, which uses the existing knowledge bases to automatically label the relation between entities within the instance. However, there is still the problem of long-tail distribution (Zhang et al., 2019) at the level of data distribution. The core problem with long-tailed distributions is the paucity of labeled instances of tail classes, which severely reduces the accuracy of the model in learning tail instance features. In this article we consider the problem of long-tailed data distribution as a problem of training with few samples. The few-shot learning is a method of training using a small number of samples, which can effectively improve the performance of tail instance relation extraction.

Currently few-shot learning is mainly applied in the field of image classification (Soudy, Afify & Badr, 2021; Fu, Zhou & Chen, 2022) to solve the training issue of lack of labeled data. In recent years, relation extraction has faced increasing challenges from the long-tail distribution of data, and few-shot learning methods have been gradually applied to relational extraction. The main purpose of the few-shot relation extraction task is to quickly learn features of relation classes using a small number of supported instances, and these features are used to predicate the relations of query instances, as shown in Table 1. Han et al. (2018) first introduced few-shot learning into relation extraction and proposed the dataset FewRel. Gao et al. (2019a) proposed an attention-based hybrid prototype network model and validated it on the dataset FewRel. In the same year, Gao et al. (2019b) released the dataset FewRel 2.0, which is designed for cross-domain challenge tasks. Thereafter, these two datasets have been widely used in few-shot relation extraction methods. Ye & Ling (2019) proposed the multi-level matching and aggregation network (MLMAN) model based on few-shot learning, which enhances the representativeness of relational prototypes by multi-level matching and aggregation of support instances with query instances. Cao et al. (2021) proposed a generic method for learning relational prototypes from unlabeled text, which extracted relations from tail instances by transferring knowledge of relational prototypes. Ding et al. (2021) improved the representation of relational prototypes with reference to the spherical coordinate system, which takes the unit vector in the unit ball as the prototype and the data set is clustered around the prototype vector on the surface of the unit sphere.

**Table 1:** Example of the 3-way 2-shot few-shot relation extraction task.

Relation	Support set
R1: Birthplace	Instance 1: Donald Trump was born in New York. Instance 2: Lin Dan was born in Longyan.
R2: School	Instance 1: Belaq Hussein Obama graduated from Harvard Law School. Instance 2: Yao Ming graduated from the University of Hong Kong.
R3: Author	Instance 1: The Count of Monte Cristo is a full-length novel written by the French writer Alexandre Dumas.
	Instance 2: Dream of the Red Chamber is written by Cao Xueqin.
**Relation**	**Query set**
R1, R2 or R3	Instance: Wuthering Heights is the work of female writer Emily Bronte.

**Note:**

The support set for this task has three relation classes, with two instances of each class. The entities in the instances are underlined and R is the relation label.

The above approaches use matching to relational prototypes to extract relations on query instances, but ignores the symmetry of the data in this process. We propose the bidirectional matching and aggregation network (BMAN) model for relation extraction. Our hypothesis is that if the relational prototype obtained from support set learning is able to extract relations on query set, then the relational prototype obtained from query set learning is also able to extract relations on the instances that are the same as the support instances relations. The reverse mechanism is designed to enable the model to learn features that are more general to the class of relation, which improves the accuracy of relation extraction for tail instances.

Generally, the main contributions of this article are summarized below:

- We propose the bidirectional matching and aggregation network that exploits the symmetry of the data in the matching process to mutually validate the prediction accuracy in both the forward and reverse, thereby improving the accuracy of the model relation extraction.
- We designed the data enhancement method to generate multiple sets of the reverse support instance using a limited number of forward query instances, while in order to avoid overfitting problem, the reverse query sets were not directly replaced by the forward support sets.
- We have conducted ample experiments on the datasets FewRel (Han et al., 2018) and FewRel 2.0 (Gao et al., 2019b), and the experimental results show that our model achieves state-of-the-art performance.

The rest of the article is structured as follows. The related work is described in Section 2. In Section 3 the bidirectional few-sample relation extraction task is defined, followed by a detailed description of the framework of the BMAN model. In Section 4 the experiments are presented and the results analyzed. Finally, in Section 5, we summaries the work and list future work.

## Related work

### Relation extraction

In recent years, the distant supervision method (Mintz et al., 2009) has been widely used in relation extraction, while achieving excellent result, which also brings the problem of noise in data annotation quality and long-tail distribution of data. For the noise problem, the introduction of the attention mechanism (Luo et al., 2018), the segmental convolutional neural networks (Li et al., 2020) and the multiple instance learning (Lin et al., 2021) have effectively improved the accuracy of automatic annotation. For the problem of long-tailed distribution of data, Zhang et al. (2019) proposed a distant supervision relation extraction method for long-tailed distribution of data, which transfers the knowledge of a large number of identified labeled instances to the relation extraction of scarcity instance classes. Subsequently, Cao et al. (2021) proposed a general method for learning relational prototypes from unlabeled text, which uses relational prototypes to transfer knowledge from head classes to tail classes, therefore improving the performance of relation extraction for tail classes.

### Few-shot relation extraction

Early few-shot learning was used in image classification tasks (Wang et al., 2019; Fu, Zhou & Chen, 2022), which aims to classify instances of a query set with a small number of samples by learning generic features of each class. Han et al. (2018) first defined relation extraction as a few-shot learning task and proposed the few-shot relation extraction dataset FewRel, which was generated using distant supervision annotation method and manual annotation method. Gao et al. (2019a) proposed the prototype network of hybrid attention that uses the instance-level attention mechanism to filter out representative support instances and focuses on the important dimensions in the feature space using the feature-level attention mechanism. In the same year, Gao et al. (2019b) proposed the FewRel 2.0 dataset with (N + 1) relation classes, which added cross-domain data and none-of-the-above data to the FewRel dataset. To strengthen the connection between relational prototypes and query sets, Ye & Ling (2019) proposed a multi-level matching and aggregation network that considers the matching between support instances and query instances at the instance level to compute relational prototypes. Ren et al. (2020) proposed the two-stage prototype network for the incremental few-shot relation extraction task, which serves to dynamically identify novel relation in support instances. Liu et al. (2021) proposes a global transformed prototype network based on the learning discriminant paradigm, which extracts relations by learning the differential information between query instances and all target classes. This capability of the model is better suited to the processing of cross-domain data. Wang et al. (2022) proposed the hybrid enhanced prototype network model which enhances the scale and utilization of annotated data.

## Methodology

In this section, we define the bidirectional few-shot relation extraction task, then describe the BMAN model framework, which is designed to extract more representative class prototype features, and finally describe the data augmentation extension method. We describe the detailed process of our method in three parts below.

### Task definition

In the few-shot learning method, the dataset is generally divided into a training set and a test set. The training set consists of the support set ( ${t_{{\rm ts}}}$) and the query set ( ${t_{{\rm tq}}}$). If there are N relations in the ( ${t_{{\rm ts}}}$) set, each containing K instances, then this few-shot learning is called the N-way K-shot task, and the label of each ( ${t_{{\rm tq}}}$) set is one of these N relation labels. The test set is also divided into the support set ( ${t_{cs}}$) and the query set ( ${t_{cq}}$) in order to follow the consistency of the test environment with the training environment. The prediction query instance relation in BMAN based on few-shot learning for forward and inverse processes can be expressed as:

(1) $$P(l|S,q) = \displaystyle{{\exp (f(\{ s_k^l\} _{k = 1}^K,q))} \over {\sum\nolimits_{i = 1}^N {\exp (f(\{ s_k^i\} _{k = 1}^K,q))} }},$$

(2) $$\hat l = \mathop {\arg \max }\limits_l {\rm P(}l|S,q{\rm )},$$

where $S$ represents the forward support set $S = \{ s_k^i;i = 1,...,N,\; k = 1,...,K\}$, $s_k^i$ is the k-th instance of class $i$, $l$ is the label of the support instance, $\hat l$ is the prediction label for the query instance, the function $f(\{ {s_k}\} _{k = 1}^K,q)$ is used to calculate the degree of match between the query instance $q$ and the support instances $\{ s_k^{}\} _{k = 1}^K$. In the training stage, we used the cross-entropy loss function which reacts to the distance between the predicted label and the true label:

(3) $${J _{forward}} = - \displaystyle{1 \over R}\sum\limits_{(q,l) \in Q}^{} {\log P{{(l|S,q)}_{forward}}},$$

(4) $${J _{reverse}} = - \displaystyle{1 \over {{R}^{\prime}}}\sum\limits_{({q}^{\prime},{l}^{\prime}) \in {Q}^{\prime}}^{} {\log P{{(l|{S}^{\prime},{q}^{\prime})}_{reverse}}},$$

where ${{\rm J}_{forward}}$ is the forward loss function, $Q$ represents the forward query set $Q = \{ ({q_j},{l_j});j = 1,...,R\}$, and R is the sample size of the forward selected query set, ${l_j} \in \{ q,...,N\}$ is the relation label of instance ${q_j}$. ${{\rm J}_{reverse}}$ is the reverse loss function ${Q}^{\prime}$ represents the reverse query set ${Q}^{\prime} = \{ ({{q}^{\prime}_j},{l_j});j = 1,...,{R}^{\prime}\}$, ${{l}^{\prime}_j} \in \{ q,...,N\}$ is the relational label of instance ${{q}^{\prime}_j}$. Combine Eqs. (3) and (4) to form the final function:

(5) $${{\rm J}_{total}} = {{\rm J}_{forward}} + \displaystyle{\alpha \over x}{{\rm J}_{reverse}},$$

where $\alpha$ is the weighting parameter, $x$ is the group number of the query set in the reverse mechanism.

In summary, the BMAN model based on few-shot learning generates N-way K-shot tasks by randomly selecting instances in the dataset, and utilizes the N-way K-shot tasks to learn features of relation classes. Next, query instances are matched with the calculated relation class features, and the relations between entities in the query instances are predicted by the matching degree.

### Bidirectional matching and aggregation network framework

**Figure 1:**
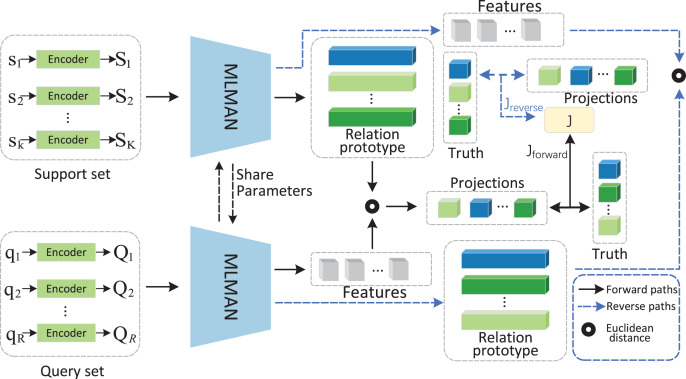
BMAN model structural framework. Operators and paths are illustrated in the dashed box in the lower right corner. The black line represents the forward matching paths, and the blue line represents the reverse matching paths. After the forward matching process, queries obtain the pseudo-label and generate reverse prototypes to perform reverse matching.

The article proposes the bidirectional matching and aggregation network based on MLMAN. The model BMAN has four main components and the framework is shown in Fig. 1.

1. Instances encoder. Bidirectional Encoder Representations from Transformer (BERT) (Devlin et al., 2018) extracts the features of the instances based on different perspectives and embeds the instance features into a vector representation.
2. Multi-level matching and aggregation. The results of the sentence encoder are fed into MLMAN to obtain a matching representation of the support instance and the query instance, then the match between the support instance and the query instance is calculated.
3. Relational prototype. The matching results obtained in the previous step are used as weights for the aggregated support instances to obtain a vector representation of the relation class.
4. Bidirectional matching. The results of the forward matching are used as the support set for the reverse matching, while the results of the reverse matching guide the forward matching, which makes the obtained relational prototypes more accurate.

#### Instances encoder

The Instance Encoder module encodes each instance $t$ into the same embedding space: $w = \kappa (t)$, where $w$ is the embedding of each instance, which will be used for matching representation learning. Existing instance encoder methods, including convolutional neural networks (CNN) (Zeng et al., 2014); recurrent neural networks (RNN) (Hochreiter & Schmidhuber, 1997); and transformer architectures (Vaswani et al., 2017). In our method, we use an instance encoder based on BERT (Devlin et al., 2018; Baldini Soares et al., 2019), which packages the entities in the instance with special tokens *ENTiTY* and */ENTiTY* and concatenates the representation of the first token *ENTiTY* of each entity as the instance embedding.

#### Matching and aggregation

After the instance encoding has obtained the embedding of the query instance $q$ and the embedding of the support instance $s$, these embedding representations are input to the matching aggregation module to compute the matching representation of the query instance with the supporting instances. The matching information between them is first calculated by the following formula

(6) $${{q}^{\prime \prime}_m} = \sum\limits_{n = 1}^{{T_s}} {\displaystyle{{\exp (q_m^{\rm T}{s_n})} \over {\sum\nolimits_{n = 1}^{{T_s}} {\exp (q_m^{\rm T}{s_n})} }}} {s_n},$$

(7) $${{s}^{\prime \prime}_n} = \sum\limits_{m = 1}^{{T_q}} {\displaystyle{{\exp (q_m^{\rm T}{s_n})} \over {\sum\nolimits_{m = 1}^{{T_q}} {\exp (q_m^{\rm T}{s_n})} }}} {q_m},$$

where $m \in \{ 1,...,{T_q}\}$, ${T_q}$ is the length of the query instance, $n \in \{ 1,...,{T_s}\}$, ${T_s}$ is the length of the query instance. Then, the matching information is fused with the original representation

(8) $$\bar Q = ReLU ([Q;{Q}^{\prime \prime};|Q - {Q}^{\prime \prime}|;Q \otimes {Q}^{\prime \prime}]{W_1}),$$

(9) $$\bar S = ReLU ([S;{S}^{\prime \prime};|S - {S}^{\prime \prime}|;S \otimes {S}^{\prime \prime}]{W_1}),$$

where $Q$ and $S$ are matrices for the original representation $q$ and $s$ aggregation, ${Q}^{\prime \prime}$ and ${S}^{\prime \prime}$ are matrices for the matching information ${q}^{\prime \prime}$ and ${s}^{\prime \prime}$ aggregation, $\otimes$ is the element-wise product and ${{\rm W}_1}$ is the weight matrix. Finally the matches are aggregated into a single vector for each query and each support instance using a max pooling together with an average pooling. The computation is as follows:

(10) $${\hat s_k} = [max({\bar S_k});ave({\bar S_k})],\forall k \in \{ 1,...,K\},$$

(11) $$\hat q = [max(\bar Q);ave(\bar Q)],$$

where ${\hat {\rm s}_k}$ is the support instance matching representation, $\hat {\rm q}$ is the query instance matching representation, ${\bar S_k}$ is a representation of K splits of $\bar S$, corresponding to K support instances.

#### Relational prototype

The query instance match representation $ \hat{\rm q}$ and the support instance match representation ${\hat {\rm s}_k}$ output by the matching and aggregation module are fed into the ReLU layer to calculate the match ${\mu _k}$ between $ \hat{\rm q}$ and ${\hat {\rm s}_k}$

(12) $${\mu _k} = {{\rm v}^{\rm T}}(ReLU({W_2}[{\hat s_k};\hat q])),$$

where ${{\rm v}^{\rm T}}$ and ${{\rm W}_{\rm 2}}$ are the parameter matrices. Finally, the matching degree ${\mu _{\rm k}}$ is used as a weight to calculate the relational prototype ${ \bar s}$ of the support instances

(13) $${ \bar {\rm s}} = \sum\limits_{k = 1}^K {\displaystyle{{\exp ({\mu _k})} \over {\sum\nolimits_{{k}^{\prime} = 1}^K {\exp ({\mu _{{k}^{\prime}}})} }}} {\hat {\rm s}_k}.$$

#### Bidirectional mechanism

The flow of the bidirectional mechanism is shown in Fig. 1. After the relational prototype ${\bar {\rm s}}$ and query instance $ \hat{\rm q}$ are calculated, the function $f(\{ {s_k}\} _{k = 1}^K,q)$ in Eq. (4) is defined as

(14) $$f{(\{ {s_k}\} _{k = 1}^K,q)_{forward}} = {v^{\rm T}}{(ReLU({W_2}[\bar s;\hat q]))_{forward}},$$

which is called forward mechanism in this article.

After identifying the labels for the forward match, the results of the forward match are mapped to the support set ${\rm {S}^{\prime}}$ to participate in the reverse mechanism. To ensure consistency in the matching calculation, the same method as forward matching is used in the reverse mechanism. The function $f(\{ {s_k}\} _{k = 1}^K,q)$ is defined in reverse matching as

(15) $$f{(\{ {{s}^{\prime}_k}\} _{k = 1}^K,{q}^{\prime})_{reverse}} = {v^{\rm T}}{(ReLU({W_2}[{\bar s}^{\prime};{\hat q}^{\prime}]))_{reverse}},$$

where ${ {\bar {\rm s}}^{\prime}}$ is the relational prototype and ${\hat {\rm q}^{\prime}}$ is the query instance in the inverse mechanism. There are two advantages to using the bidirectional mechanism: (1) Information from symmetric data can be obtained to improve the accuracy of model relation extraction. (2) The positive and negative labeled samples generated by the forward mechanism can be used to obtain a more accurate relational prototype.

### Data enhancement method

**Figure 2:**
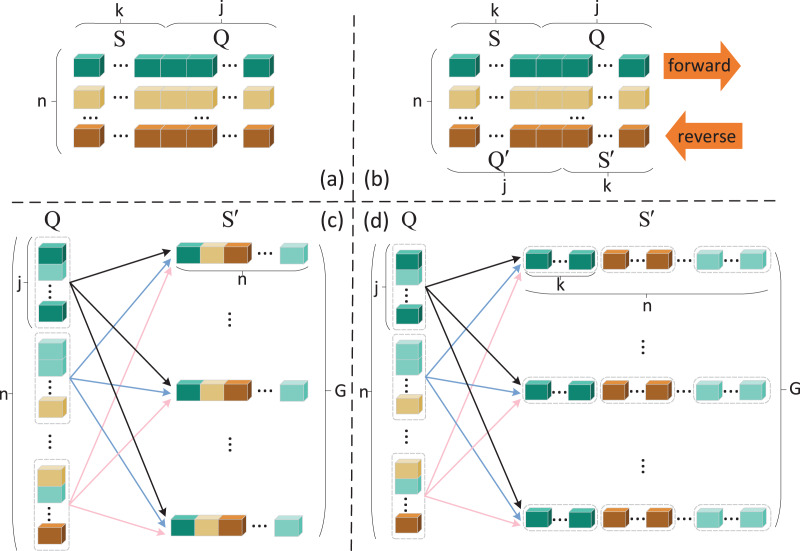
Data enhancement method design process. Squares of the same color represent instances of the same relation. (A) Single direction method (B) BMAN- simple, (C) BMAN- link, (D) BMAN.

The conversion of forward and reverse data in the framework utilizes data enhancement method, which is responsible for generating the reverse support set ${\rm {S}^{\prime}}$ by the forward query set ${\rm Q}$ and generating the reverse query set ${\rm {Q}^{\prime}}$. The design of this method is a simple to complex process. The reverse support set ${\rm {S}^{\prime}}$ and the reverse query set ${\rm {Q}^{\prime}}$ are divided directly on the single direction queue of instance sets, which is called BMAN-simple, as shown in Fig. 2. But such an approach does not exploit the results of forward matching and severs the connection between forward and reverse in the matching and aggregation network, which is not the result we expected. To address the above shortcoming, we use the forward matching results to recombine the instances in the query set ${\rm Q}$ to obtain multiple reverse support sets ${\rm {S}^{\prime}}$, as shown in Fig. 2, and randomly select instances of the same class as the support set S to form the reverse query set ${\rm {Q}^{\prime}}$, which is called BMAN-link. We also found that the labels of the m consecutive instances in the k-shot task were identical, so that the logic of BMAN-link was not applicable to the k-shot task. This article also improves on BMAN-link to form the final BMAN model, which uses the 1-shot task as a special k-shot, as shown in Fig. 2. In order to ensure that the reverse support set ${\rm {S}^{\prime}}$ contains instances of n relation classes, this article devises solutions for two possible scenarios:

1. Reuse instances within instance-poor classes when the distribution of forward matching results is unbalanced.
2. When instances of a certain class are completely missing, instances of this class in the forward support set S are introduced.

This method maintains the range of relation classes while expanding the size of the instances, which allows the model to obtain more information about the relational prototypes while avoiding overfitting problem. The specific code implementation is shown in Algorithm 1.

**Algorithm 1:** Generating support sets and query sets for the reverse mechanism. $M$ is a dataset holding instances of the same relation class. ${{\rm Q}_l}$ and ${{\rm T}_l}$ represent the query set and training set of the label $l$ respectively. $x$ is the number of groups in the reverse mechanism, initially 0. ${b_x}$ is the number of positive samples in each set of reverse matching support set ${{\rm {S}^{\prime}}_x}$.

**Input:** (1) Forward query set ${\rm Q}$. (2) Support Set ${\rm S}$. (3) Training set ${\rm T}$. (4) Task scenario n-way k-shot.
**Output:** Reverse support set ${\rm {S}^{\prime}}$ and reverse query set ${\rm {Q}^{\prime}}$. Parameters $x$ and $\alpha$.
**for** k = 1 **do**
${M_i} \leftarrow SAMESAMPLE({q_j},{{\rm Q}_{\rm l}})$
$x + +$
if x < k ∇ Reusing instances from a dataset $M$ with few instances
$({q_{j + 1}},{M_i}) \leftarrow ({q_j},{M_i})$
if i < n
${M_i} \leftarrow SAMESAMPLE({q_j},{\rm S})$
${{\rm {S}^{\prime}}_x} \leftarrow DIFFERENTSAMPLE({M_i})$
${\alpha _x} = \displaystyle{{{b_x}} \over R}$
${{\rm {Q}^{\prime}}_x} \leftarrow RANDOMSAMPIE(q,{T_l})$
** return** ${{\rm {S}^{\prime}}_x}{\rm ,}{{\rm {Q}^{\prime}}_x},{\alpha _x},x$
**end for**
**for** k > 1 **do**
${M_i} \leftarrow SAMESAMPLE({q_j},{{\rm Q}_l})$
$x + +$
if $x$ < k
$({q_{j + 1}},M) \leftarrow ({q_j},M)$
if $i$ < n
${M_i} \leftarrow SAMESAMPLE({q_j},{\rm S})$
∇ Select ${\rm k}$ instances of each $M$ and place them in the support set ${{\rm {S}^{\prime}}_x}$.
${{\rm {S}^{\prime}}_x} \leftarrow DIFFERENTSAMPLE({M_i},k)$
${\alpha _x} = \displaystyle{{{b_x}} \over R}$
${{\rm {Q}^{\prime}}_x} \leftarrow RANDOMSAMPIE(q,{T_l})$
** return** ${{\rm {S}^{\prime}}_x},{{\rm {Q}^{\prime}}_x},{\alpha _x},x$
**end for**

## Experiments

In this section, the BMAN model is compared with existing baseline methods to show the advantages of this approach.

### Dataset

This article evaluates the BMAN model on two publicly available datasets, details of which are shown in Table 2. The dataset FewRel (Han et al., 2018) is the first proposed dataset for few-shot relation extraction, which is based on Wikipedia text and constructed using distant supervised learning and manual annotation, with the data distribution obeying a balanced distribution. The dataset FewRel contains 100 relations with 700 instances each, where the train set contains 64 relation classes, the validation set contains 16 relation classes and the test set contains 20 relation classes. The dataset FewRel 2.0 (Gao et al., 2019b) adds cross-domain data based on dataset FewRel which introduces biomedical texts from the PubMed dataset and UMLS dataset to generate the test set, where 25 relation classes with 100 instances each are included, and 17 relations from the SemEval dataset are selected to construct the validation set. The datasets FewRel 2.0 and FewRel share the same train set.

**Table 2:** Datasets FewRel and FewRel 2.0.

Dstaset	Apply	Source	Relation number	Instances number
FewRel	Train	Wiki	64	44,800
	Val	Wiki	16	11,200
	Test	Wiki	20	14,000
FewRel 2.0	Train	Wiki	64	44,800
	Val	SemEval	17	8,851
	Test	PubMed	25	2,500

### Local verification

#### Hyperparameter

**Figure 3:**
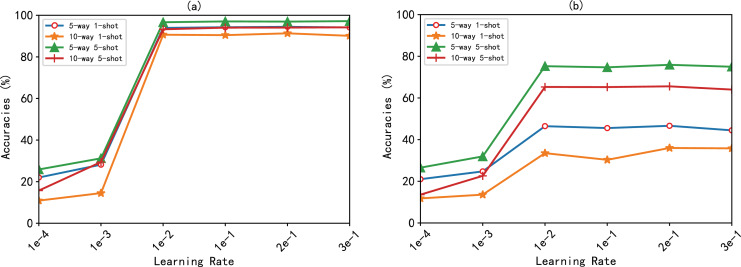
Accuracy (%) of the BMAN at different learning rates. (A) Accuracy of the BMAN in the FewRel dataset. (B) Accuracy of the BMAN in the FewRel 2.0 dataset.

The effect of hyperparameters in the model was experimentally investigated, with the model trained on a device with an NVIDIA RTX3090 24G GPU. Due to GPU video memory limitations, the experiments were set up with a query set sample of $R = 5 \cdot n$, where $n$ is the number of total relation classes, and the value in the learning rate $learn\_rate \in (1e - 4,1e - 3,1e - 2,2e - 2,1{\rm e} - 1,2e - 1,3e - 1)$ that makes the model accurate is determined, as shown in Fig. 3. As can be seen from the figure, on the FewRel, FewRel 2.0 dataset. The model BMAN obtained the highest accuracy when the learning rate was 0.2.

#### Data enhancement method

We compare the accuracy of BMAN-simple, BMAN-link and BMAN on datasets FewRel and FewRel 2.0, as shown in Table 3. Table 3 shows that the overall accuracy of the BMAN model is the best, while the overall accuracy of the BMAN-simple model is poor. The BMAN-simple model severs the connection between the forward and the reverse which does not serve the purpose of extracting symmetric information from the data. The BMAN-link model achieved the best results in the 1-shot task, but the worst accuracy in the k-shot task, which we analyzed because the overall logic of the k-shot task was broken by 1-shot task as the reverse matching of the k-shot task. The BMAN model considers the difference between 1-shot and k-shot tasks which strengthens the connection between the reverse mechanism and the forward mechanism, while maintaining the consistency between the forward matching logic and the reverse matching logic.

**Table 3:** Accuracy (%) of BMAN-simple, BMAN-link and BMAN in datasets FewRel and FewRel 2.0.

Model	Datasets	5-way 1-shot	5-way 5-shot	10-way 1-shot	10-way 5-shot
BMAN-simple	FewRel	89.09	96.96	82.92	94.23
	FewRel2.0	45.18	73.83	32.20	63.89
BMAN-link	FewRel	**95.12**	20.94	**92.37**	11.14
	FewRel2.0	**49.12**	20.32	**37.87**	10.98
BMAN	FewRel	**95.12**	**98.17**	**92.37**	**95.18**
	FewRel2.0	**49.12**	**77.54**	**37.87**	**68.64**

**Note:**

Values with the highest accuracy in the same task are shown in bold.

### Experimental results and analysis

**Figure 4:**
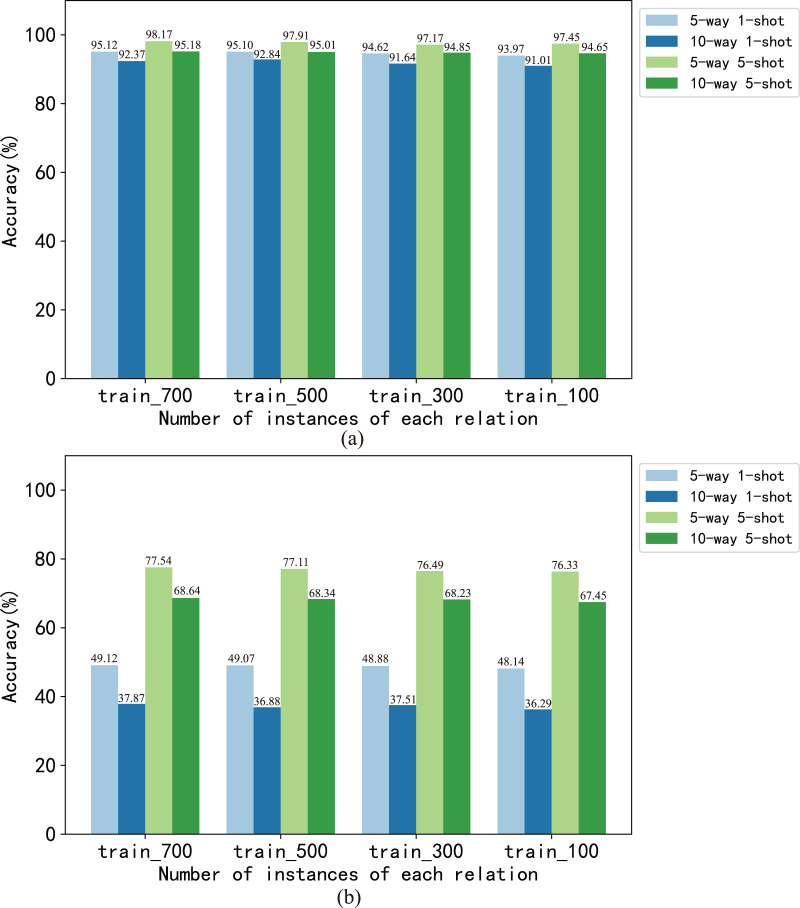
Accuracy (%) of model BMAN on different number of instances. (A) Accuracy in vertical domain dataset FewRel. (B) Accuracy in cross-domain dataset FewRel 2.0.

**Figure 5:**
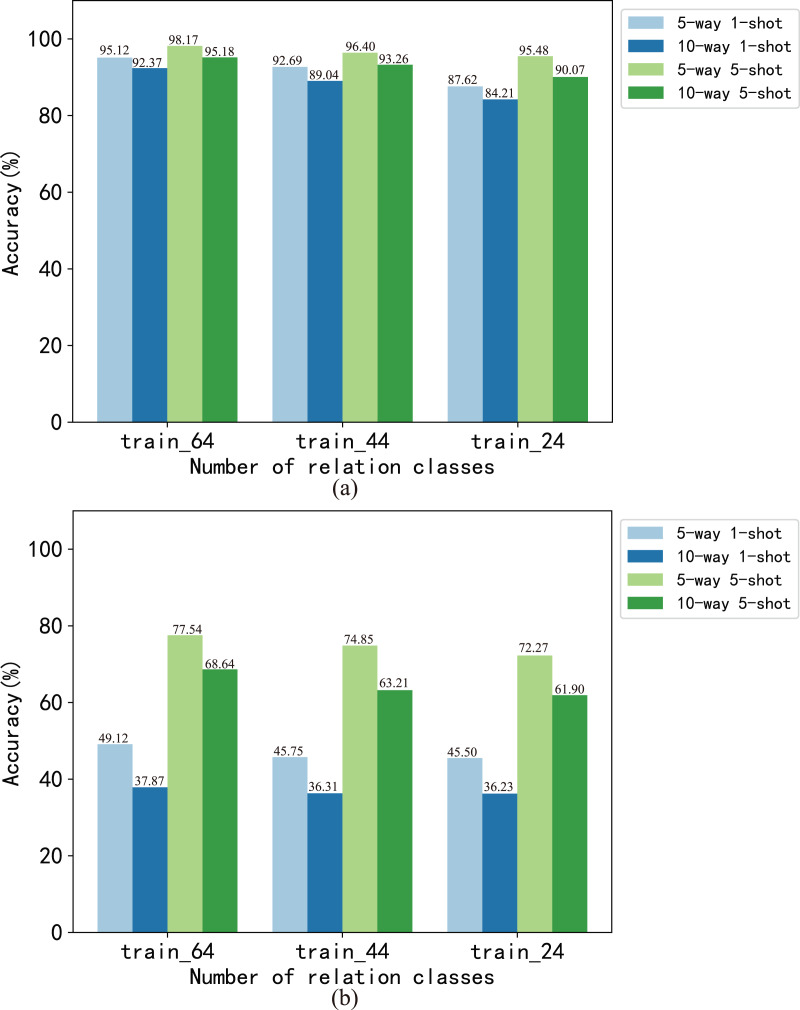
Accuracy (%) of model BMAN on different number of relations. (A) Accuracy in vertical domain dataset FewRel. (B) Accuracy in cross-domain dataset FewRel 2.0.

In this article, we analyzed the effect of the number of instances and relations on model performance at the dataset level and task level respectively. We set the number of instances of each relation in the train set to 700, 500, 300 and 100, respectively, which generated four training sets of train_700, train_500, train_300 and train_100. The BMAN model was trained on these train sets, and the results are shown in Fig. 4. The results show that the accuracy of the 5-way 1-shot task fluctuates within 1.15 on the FewRel dataset and within 1.24 on the FewRel 2.0 dataset. The accuracy of the 10-way 1-shot task fluctuates within 1.61 on the FewRel dataset and within 1.58 on the FewRel 2.0 dataset. The accuracy of the 5-way 5-shot task fluctuates within 0.72 on the FewRel dataset and within 1.21 on the FewRel 2.0 dataset. The accuracy of the 10-way 5-shot task fluctuates within 0.53 on the FewRel dataset and within 1.19 on the FewRel 2.0 dataset. We set the number of relations in the train set to 64, 44 and 24, respectively, which generated three train sets of train_64, train_44 and train_24 for verifying the effect of the number of relations on BMAN, and the results are shown in Fig. 5. The results show that the accuracy of the five-way one-shot task fluctuates within 7.5 on the FewRel dataset and within 3.62 on the FewRel 2.0 dataset. The accuracy of the 10-way one-shot task fluctuates within 8.16 on the FewRel dataset and within 1.64 on the FewRel 2.0 dataset. The accuracy of the five-way five-shot task fluctuates within 2.69 on the FewRel dataset and within 5.27 on the FewRel 2.0 dataset. The accuracy of the 10-way five-shot task fluctuates within 5.11on the FewRel dataset and within 6.74 on the FewRel 2.0 dataset. The results of the above two experiments show that the number of instances have a weak effect on BMAN performance and the number of relations have a strong effect on BMAN performance at the dataset level, which indicates that BMAN can be used in scenarios with sufficient relations and few instances, and can effectively solve the problem of lack of tail instances in the long-tail distribution of data. The experimental results also show that the performance of the BMAN model decreases with increasing ‘*way*’ and increases with increasing ‘*shot*’ at the task level, which means that the performance of the model decreases as the number of relations in the task increases and increases as the number of instances selected for the task increases.

**Figure 6:**
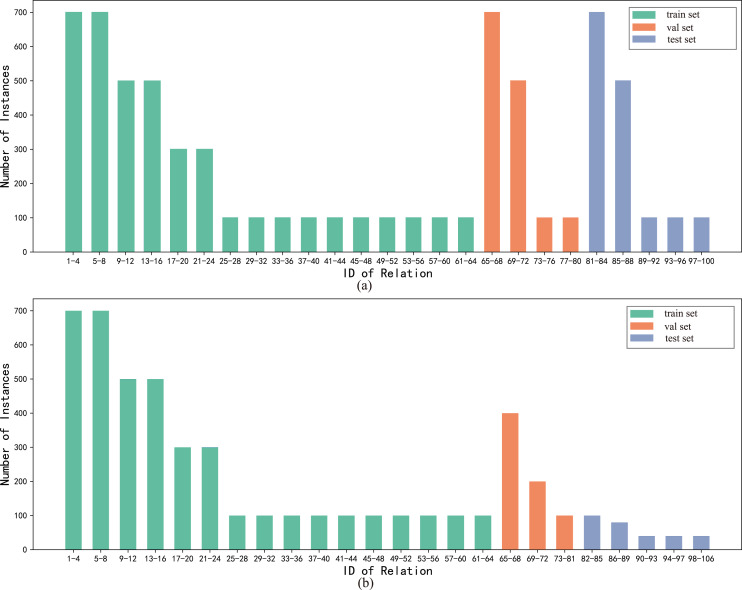
The division of the train, validation and test sets in the datasets FewRel_L and FewRel 2.0_L, and the distribution of the number of instances. The horizontal coordinates n-m indicate the relation class labels n, n + 1, …, m and the vertical coordinate is the number of instances each relation. (A) The division of the training set, validation set, test set and the distribution of the number of instances in FewRel_L. (B) The division of the training set, validation set, test set and the distribution of the number of instances in FewRel 2.0_L.

To directly validate the performance of the model on the long-tailed distribution datasets, we constructed the long-tailed distribution datasets FewRel_L and FewRel 2.0_L based on random sampling of the original datasets, where the relation classes of the train, validation and test sets were kept constant and the number of instances was adjusted, as shown in Fig. 6. The datasets FewRel_L and FewRel 2.0_L share the same training set and the validation results of the BMAN model are shown in Table 4. The accuracy of the BMAN model on the datasets FewRel_L for the tasks five-way one-shot, 10-way one-shot, five-way five-shot and 10-way five-shot lost 0.15, 0.94, 0.84 and 0.38 respectively. The accuracy of the BMAN model lost 0.23, 1.02, 1.13, and 0.39 on the datasets FewRel 2.0_L for the tasks five-way one-shot, 10-way one-shot, five-way five-shot, and 10-way five-shot respectively. The results show that the model BMAN keeps excellent performance on the long-tailed distribution dataset, which indicates that the model BMAN is effective for solving the long-tailed distribution of the data.

**Table 4:** Accuracy (%) of BMAN on datasets FewRel, FewRel 2.0, FewRel_L and FewRel 2.0_L.

Datasets	5-way 1-shot	5-way 5-shot	10-way 1-shot	10-way 5-shot
FewRel_L	94.97	97.33	91.43	94.80
FewRel2.0_ L	48.89	76.41	36.85	68.25
FewRel	95.12	98.17	92.37	95.18
FewRel2.0	49.12	77.54	37.87	68.64

**Figure 7:**
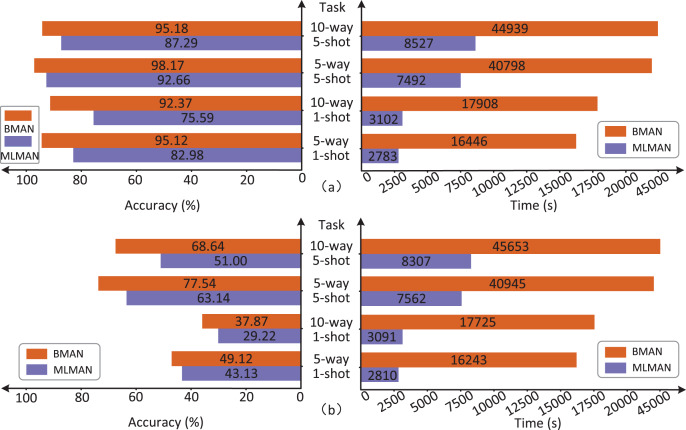
Comparison of the accuracy and time complexity between BMAN and MLMAN. (A) Comparison results in the dataset FewRel. (B) Comparison results in dataset FewRel2.0.

Since both the BMAN and the MLMAN use matching and aggregation network, this article evaluates the time complexity of the BMAN for different task scenarios using MLMAN as the baseline, as shown in Fig. 7.

The comparison in the Fig. 7 shows that the accuracy of the BMAN model improves which comes at the cost of time complexity. In the datasets FewRel and FewRel2.0, the accuracy improvements in the five-way one-shot task were 14.63% and 13.89% respectively, while the corresponding time complexity improvements were 490.95 and 478.04% respectively. The accuracy improvements in the 10-way one-shot task were 22.20% and 29.60% respectively, while the corresponding time complexity improvements were 477.30% and 473.44% respectively. The accuracy improvements in the five-way five-shot task were 5.95% and 22.81% respectively, while the corresponding time complexity improvements were 444.55% and 441.46% respectively. The accuracy improvements in the 10-way five-shot task were 9.04% and 34.59% respectively, while the corresponding time complexity improvements were 427.02% and 449.57% respectively. The increase in time complexity is due to the fact that a set of forward query set can be mapped to multiple sets of reverse support set during the bidirectional training process with increased computational effort for reverse matching.

This article also selects methods as a baseline for comparison and evaluates the performance of the BMAN model for relation extraction in vertical domain. The evaluation metrics in this article follow the settings in FewRel and include accuracy on the five-way one-shot, five-way five-shot, 10-way one-shot and 10-way five-shot tasks. This article compares the BMAN with the following baselines:

- MLMAN (Ye & Ling, 2019): This approach interactively encodes support instances with query instances, and the matching process strengthens the connection between the query set and the relational prototype.
- CTEG (Wang et al., 2020): The model uses entity-guided attention mechanisms and confusion-aware training to distinguish between problems of relational confusion.
- TPN(BERT) (Wen et al., 2021): The method integrates the transformer model into the prototype network and uses the pre-trained BERT as the encoder for the model.
- ConceptFERE (Yang et al., 2021): The model enhances the influence of entity concepts on relations in relation extraction.
- COL Final (Ding et al., 2021): The method learns the relational prototype from contextual information, which focuses on the influence of semantics on the relational prototype, and uses spherical coordinates as the basis for prototype interpretation.
- GTPN (Liu et al., 2021): The model is based on the learning-to-discriminate paradigm and focuses more on the discriminatory knowledge between all candidate classes.

As can be seen from Table 5, BMAN achieved the highest accuracy on the FewRel dataset compared to previous studies. BMAN compared to COL Fina respectively improved by 2.1% and 5.71% on the five-way one-shot and 10-way one-shot tasks. BMAN compared to GTPN respectively improved by 0.24% and by 0.46% on the five-way five-shot and 10-way five-shot tasks. It is proven that the bidirectional matching and aggregation mechanism can effectively improve the accuracy of relation extraction.

**Table 5:** Accuracy (%) of the different models on the FewRel dataset.

Models	No.	5-way 1-shot	5-way 5-shot	10-way 1-shot	10-way 5-shot
BMAN	1	**95.12**	**98.17**	**92.37**	**95.18**
MLMAN (2019)	2	82.98	92.66	75.59	87.29
CTEG (2020)	3	84.72	92.52	76.01	84.89
TPN (BERT) (2021)	4	80.14	93.60	72.67	89.83
ConceptFERE (2021)	5	89.21	–	75.72	–
COL Final (2021)	6	**92.51**	95.88	**86.39**	92.76
GTPN (2021)	7	89.40	**97.00**	84.40	**93.80**

**Note:**

The highest accuracy values of the comparison model in the same task scenario and the accuracy values of the BMAN model are in bold.

We also conducted comparative experiments on the cross-domain dataset FewRel2.0 to evaluate the performance of the BMAN model. We chose the baseline methods for the comparison, which used FewRel2.0 as the dataset:

- Proto-Bert (Gao et al., 2019b): Bert is utilized as an encoder in the prototype network.
- Proto-CNN (Gao et al., 2019b): CNN is utilized as an encoder in the prototype network.
- Proto-Adv (Bert) (Gao et al., 2019b): Bert is utilized as an encoder in the prototype network for adversarial training.
- Proto-Adv (CNN) (Gao et al., 2019b): CNN is utilized as an encoder in the prototype network for adversarial training.

Table 6 shows that BMAN achieves the best performance in these tasks compared to other approaches based on the learning-to-matching paradigm, but with a significant difference compared to GTPN. The reason for this result is that the BMAN model focuses more on matching between relational prototype features and query instance features, whereas GTPN focuses more on discriminating between different features of query instances and target relation classes, which is more important for relation extraction of cross-domain data. Specifically, the main advantage of GTPN is that all candidate relation classes are considered jointly rather than independently when extracting relations, which allows the model to more accurately use discriminative information between classes to distinguish between relation confusions caused by cross-domains. In contrast, the BMAN model has a larger error in measuring the degree of similarity between query instances and out-of-domain relations in the cross-domain case. However, the measurement of query instance-prototype similarity directly affects the accuracy of relation extraction within the domain, and BMAN’s bidirectional matching and aggregation enhances the measurement of query instance-prototype similarity. Thus, we proposed the BMAN model that outperformed the GTPN model in the dataset FewRel, while the GTPN model outperformed the BMAN model in the dataset FewRel 2.0.

**Table 6:** Accuracy (%) of different models on the FewRel 2.0 dataset.

Models	No.	5-way 1-shot	5-way 5-shot	10-way 1-shot	10-way 5-shot
BMAN	1	**49.12**	**77.54**	**37.87**	**68.64**
Proto-CNN (2019)	2	35.10	49.40	23.00	35.20
Proto-Bert (2019)	3	40.10	51.50	26.50	36.90
Proto-Adv (Bert) (2019)	4	41.90	54.70	27.40	37.40
Proto-Adv (CNN) (2019)	5	42.20	58.70	28.90	44.40
TPN (2021)	6	38.22	48.55	26.74	35.40
GTPN (2021)	7	**80.00**	**92.60**	**62.25**	**86.90**

**Note:**

The highest accuracy values of the comparison model in the same task scenario and the accuracy values of the BMAN model are in bold.

## Conclusions

This article proposes the BMAN based on few-shot learning. The model obtains symmetric information from the data by the bidirectional mechanism which is used to validate the relational prototype and adjust the model parameters. At the same time, the data enhancement approach provides the data scale for the model to fully exploit the information in the query set. Compared to other baseline methods, the model BMAN achieves state-of-the-art performance in vertical domains, while also being highly competitive across domains. In addition, we evaluated the time complexity of BMAN and concluded that the increased time complexity was acceptable for the extent of the performance improvement.

As future work, we consider the deficiencies of the BMAN model in cross-domain scenarios and are prepared to introduce information on the differences between relation classes to enhance the model’s discrimination of out-of-domain relations. Also, our model has a high time complexity and we consider reducing the time complexity by speeding up the convergence of the model.

## Supplemental Information

10.7717/peerj-cs.1272/supp-1 Supplemental Information 1 Code for BMAN.

## Additional Information and Declarations

### Data Availability

The following information was supplied regarding data availability: The code is available at GitHub: https://github.com/WeNg1593235091/BMAN/tree/v1.0. DOI 10.5281/zenodo.7108639. The datasets FewRel and FewRel 2.0 are available at GitHub and ACL Anthology: https://github.com/thunlp/FewRel. Han X, Zhu H, Yu P, Wang Z, Yao Y, Liu Z, Sun M. 2018. FewRel: A Large-Scale Supervised Few-Shot Relation Classification Dataset with State-of-the-Art Evaluation. DOI: 10.18653/v1/D18-1514. Gao T, Han X, Zhu H, Liu Z, Li P, Sun M, Zhou J. 2019b. FewRel 2.0: Towards More Challenging Few-Shot Relation Classification. DOI: 10.18653/v1/D19-1649.
